# Dopamine D_4_ receptors inhibit proliferation and migration of vascular smooth muscle cells induced by insulin via down-regulation of insulin receptor expression

**DOI:** 10.1186/1475-2840-13-97

**Published:** 2014-06-02

**Authors:** Changqing Yu, Zhen Wang, Yu Han, Yukai Liu, Wei Eric Wang, Caiyu Chen, Hongyong Wang, Pedro A Jose, Chunyu Zeng

**Affiliations:** 1 Department of Cardiology, Daping Hospital, The Third Military Medical University, Chongqing, P.R. China; 2 Chongqing Institute of Cardiology, Chongqing, P.R. China; 3 Division of Nephrology, Department of Medicine, University of Maryland School of Medicine, Baltimore, MD, USA; 4 Department of Physiology, University of Maryland School of Medicine, Baltimore, MD, USA

**Keywords:** Dopamine receptor, Insulin receptor, Vascular smooth muscle cell, Proliferation, Migration, Atherosclerosis

## Abstract

Vascular smooth muscle cells (VSMCs) proliferation and migration, which are central in the development of vascular diseases, are regulated by numerous hormones and humoral factors. Activation of the insulin receptor stimulates VSMCs proliferation while dopamine receptors, via D_1_ and D_3_ receptors, inhibit the stimulatory effects of norepinephrine on VSMCs proliferation. We hypothesize that activation of the D_4_ dopamine receptor may also inhibit the proliferation and migration of VSMCs, therefore, inhibit atherosclerosis. Our current study found that insulin increased the proliferation and migration of A10 cells, an effect that was reduced in the presence of a D_4_ receptor agonist, PD168077. The negative effect of the D_4_ receptor on insulin’s action may be via decreasing insulin receptor expression, because activation of the D_4_ receptor inhibited insulin receptor protein and mRNA expressions, indicating that the regulation occured at the transcriptional or post-transcriptional levels. To determine whether or not the inhibition of D_4_ receptor on insulin-mediated proliferation and migration of VSMCs has physiological significance, hyper-insulinemic Sprague–Dawley rats with balloon-injured carotid artery were treated with a D_4_ agonist, PD168077, (6 mg/kg/d) for 14 days. We found that PD168077 significantly inhibited neointimal formation by inhibition of VSMC proliferation. This study suggests that activation of the D_4_ receptor suppresses the proliferation and migration of VSMCs, therefore, inhibit atherosclerosis. The D_4_ receptor may be a potential therapeutic target to reduce the effects of insulin on artery remodeling.

## Introduction

The abnormal proliferation and migration of vascular smooth muscle cells (VSMCs) play a crucial role in neointimal formation and vascular remodeling during atherosclerosis and restenosis [1-3]. It is currently accepted that proliferation and migration of medial VSMCs are involved in neointimal formation after injury, which could be induced by cytokines and growth factors, including insulin. Insulin initiates many biological effects by activating the insulin signaling pathways, such as mitogen activated protein kinase, triggering hypertrophy, proliferation, and migration of VSMCs [4-6]. Therefore, inhibition of insulin-mediated VSMCs proliferation and migration would be helpful in preventing the development of atherosclerosis and restenosis after angioplasty.

Dopamine receptors exert beneficial effects by regulating epithelial sodium transport and vascular smooth muscle tone in hypertension [7-12]. Dopamine receptors are classified into D_1_-like and D_2_-like subtypes based on their structure and pharmacology. D_1_-like receptors are composed of D_1_ and D_5_ receptors while D_2_-like receptors are composed of D_2_, D_3_ and D_4_ receptors [6-11]. We have previously shown that activation of D_1_-like or D_3_ receptor inhibits the insulin-mediated VSMCs proliferation [13,14]. Activation of D_1_-like and D_3_ receptors has an additive inhibitory effect on norepinephrine-mediated proliferation of VSMCs [15]. We hypothesized that D_4_ receptors may also have inhibitory effect on insulin-mediated VSMCs proliferation and migration, which may inhibit the formation of atherosclerosis. To test this hypothesis, we used cultured rat aortic smooth muscle cells line (A10) to examine the potential effect of D_4_ receptors on insulin-mediated proliferation and migration, and investigated the role of D_4_ receptors on neointimal hyperplasia in Sprague–Dawley (SD) rats with hyperinsulinemia.

## Methods

### Materials

PD168077 (D_4_ receptor agonist), L745870 (D_4_ receptor antagonist), streptozotocin (STZ) were from Sigma Co. (Sigma, St. Louis, MO). Insulin was purchased from Roche Group (Basel, Switzerland); Rabbit polyclonal antibody against insulin receptor, cleaved caspase 3 and Histone H3 were from Cell Signaling (Beverly, MA). Antibody for proliferating cell nuclear antigen (PCNA) was from Santa Cruz. SDS-polyacrylamide gels were from Pierce (Rockford, IL). Polyvinylidene fluoride (PVDF) and protein gel apparatus were from Bio-Rad (Hercules, CA). Minimal essential medium (MEM), Dulbecco’s modified Eagle’s medium (DMEM), and fetal bovine serum (FBS) were from Gibco/Invitrogen (Carlsbad, CA); fibroblast growth factor (FGF), epidermal growth factor (EGF), phosphate buffered saline (PBS), penicillin/streptomycin, and non-essential amino acids were from Sigma Co.

### Cell culture

A10 cell [13], a smooth muscle cell line from rat thoracic aorta, was purchased from ATCC (A10; ATCC, Hercules, CA), and cultured in DMEM supplemented with 20% FBS and 1% penicillin/streptomycin/bFGF/EGF at 37°C in a humidified, 5% CO_2_ atmosphere. After reaching sub-confluence, the A10 cells were serum-starved for 24 hrs in serum-free DMEM to maintain quiescence before further treatment.

### A10 cell proliferation

Cell proliferation was determined by measuring the uptake of tetrazolium salt, 3-(4,5-dimethylthiazol-2-yl)-2,5diphenyltetrazolium bromide (MTT) and the incorporation of [^3^H]-thymidine (Atomic Energy Research Establishment of China, Beijing City, China) into DNA of cells respectively. The cells were seeded into 96-well (100 μl of medium per well) culture plates at a density of 1 × 10^4^ cells/well, made quiescent for 24 hrs, and then stimulated with the indicated reagents. Subsequently, 20 μl of MTT (5 mg/ml) were added to each well, and the incubation continued for an additional 4 hrs at 37°C. Thereafter, dimethyl sulfoxide (DMSO, 150 μl) was added to each well, and absorbance read at 490 nm on a Microplate reader (model 680, Bio-Rad). For [^3^H]-thymidine incorporation assay, the cells were treated with the same reagents then labeled with [^3^H]-thymidine (1 μCi/ml) 6 h and assessed for [^3^H] incorporation into newly synthesized DNA as previously described [13]. Cell number was also used to estimate cell proliferation by plating the A10 cells in six-well plastic culture dishes at a density of 1 × 10^4^/well in DMEM with 10% FBS. The medium was then removed and washed three times with PBS. The cells were incubated in serum-free medium, which contained the indicated reagents for the indicated times. After incubation, the number of cells was counted by a hemocytometer (trypan blue uptake, which indicates cell death, was observed in <10% of the cells). Experiments were performed independently at least three times [13-15].

### A10 cell migration

Cells migration was examined using transwell and scratch-wound migration assays. The transwell migration assay was performed using 24-well tissue culture plates (BD Bioscience, Becton, NJ) with an 8-μm-pore polycarbonate membrane [16,17]. The number of migratory A10 cells was counted in 10 randomly chosen fields of duplicate chambers at a magnification of 200× for each sample. For the scratch-wound migration assay, the A10 cells were scratched with a small tip along the ruler. After washing, the cells were cultured in serum-free DMEM for 48 hrs. The migration area (%) was analyzed in 10 randomly chosen fields under an inverted microscope (IX-70; Olympus, Tokyo, Japan), using NIH Image J software; area at 0 hr and area at 48 hrs × 100% was calculated.

### Immunoflurescence image of rat thoracic aorta

Expression of D_4_ receptors in thoracic aorta from SD rats was examined by laser scanning confocal microscope. Briefly, the aorta was cleared of blood with ice-cold oxygenated saline and kept in Histochoice (Amresco, Solon, OH) for one to two days at 4°C, then sectioned (4 μm), embedded in paraffin, and mounted on slides. The sections were double-immunostained with mouse anti-D_4_ receptor antibody (1:100) and rabbit polyclonal anti-α-SM-actin antibody (1:100). The colocalization of D_4_ receptors and α-SM-actin was performed as described previously [18].

### Immunoblotting

A10 cells were treated with vehicle (ddH_2_O), D_4_ receptor agonist (PD168077) and/or D_4_ receptor antagonist (L745870) at the indicated concentrations and times. After treatment, the A10 cells were washed once in PBS and lysed in lysis buffer. Immunoblotting was performed as previously reported [13,14]. Protein concentration was determined using a protein assay kit (Bio-Rad Laboratories, Hercules, CA) with bovine serum albumin as standard. Cell lysates were boiled in sample buffer (35 mmol/L Tris–HCl, pH 6.8, 4% SDS, 9.3% dithiothreitol, 0.01% bromophenol blue, 30% glycerol) for 10 min; 50 μg of cell protein were subject to immunoblotting analysis. PVDF membranes were blocked with 5% nonfat dry milk in PBS-T [0.05% Tween20 in 10 mmol/L phosphate-buffered (isotonic) saline] at 37°C for 2 hrs with constant shaking. The membranes were then probed with rabbit polyclonal antibodies specific for insulin receptor and D_4_ receptor at 4°C overnight, and then incubated with goat anti-rabbit polyclonal antibodies conjugated to horseradish peroxidase. Proteins were detected using enhanced chemiluminescence reagents (Amersham, Little Chalfont, UK). The amount of protein transferred onto the membranes was normalized by immunoblotting of α-actin (monoclonal α-actin antibody, 1:400, Santa Cruz Biotechnology, Inc. Santa Cruz, CA).

### Reverse transcriptase-PCR

Total RNA from A10 cells was isolated using a Trizol procedure (Invitrogen, Carlsbuel, CA). Two μg of total RNA were used to synthesize cDNA, which served as template for the amplification of D_4_ receptor, insulin receptor, and β-actin (as housekeeping gene). Primer sequences for D_4_ receptor were 5^′^-TGC CCT GTC CGC TCA TGC TAC TGC-3^′^ (forward) and 5^′^-CAC CGG CAG GAC TCT CAT CGC CTT GC-3^′^ (reverse). Primer sequences for insulin receptor were 5^′^-GGA CTGAAG GTA TGA ATG GAG-3^′^ (forward) and 5^′^-TAA CAC AAG CCA AGG AAG GG-3^′^ (reverse), Primer sequences for β-actin was 5^′^-GTGGGTATGGGTCAGAAGGA-3^′^(forward) and 5^′^-AGCGCGTAACCCTCATAGAT-3^′^ (reverse). The amplification was performed with the following conditions: 95°C for 1 min, followed by 35 cycles of denaturation at 94°C for 30 sec, annealing at 60°C for 30 sec, and extension at 72°C for 45 sec. This was followed by a final extension at 72°C for 10 min. The PCR products were electrophoresed in 2% agarose gels [13,14].

### Rat hyperinsulinemic model

Healthy male SD rats weighing 210-220 g, were obtained from the Experimental Animal Center of Daping Hospital. The rats were housed on a constant 12 hrs light/12 hrs dark cycle in temperature-controlled central facility (18-22°C) and allowed free access to normal chow and tap water. Type 2 diabetes was induced using STZ, as described previously [19,20]. Briefly, the rats were intragastrically fed high-fat diet (HFD) consisting of 15% lard, 30% sucrose, 10% Tween-80 and 10% propylene glycol in a volume of 20 ml/kg body weight once a day for 4 weeks (normal-pellet diet rats served as the control). Then the rats were injected intraperitoneally with STZ (25 mg/kg in a 0.1 mol/L citrate buffer at a single dose, pH 4.5) to induce type 2 diabetes with hyperinsulinemia and insulin resistance. After STZ injection, body mass and blood pressure were measured; blood samples were obtained from the tail vein for assay of blood glucose, serum insulin, triglycerides, cholesterol, and free fatty acids using commercially available kits. The rats with fasting blood glucose concentrations more than 11.1 mmol/L were considered to qualify for subsequent experiments.

### Rat carotid balloon injury

SD rats were anesthetized using a mixture of ketamine (80 mg/kg) and xylazine (12 mg/kg), and then the left common and external carotid arteries were exposed and isolated. A 2 F Fogarty catheter (Edwards Life Sciences, Irvine, CA) was introduced into the common carotid artery through an arteriotomy in the external carotid artery and inflated to 1.0-1.5 atm. A 10-mm injury was induced by withdrawing the catheter 4 times. The external carotid artery was then untied, and blood flow was restored. After the balloon-injury, SD rats were intraperitoneally injected with PD168077 (6 mg/kg per day) or similar volume of distilled water (2 ml/kg) daily for 14 days. Before the SD rats were euthanized, body mass and blood pressure were measured, and bloods for biochemical parameter measurements were obtained. Then, both carotid arteries were dissected and the sections were stained with hematoxylin and eosin (H&E) and observed under a light microscope (Olympus, Tokyo, Japan). Media and neointima areas of injured arteries were quantified by planimetry (μm^2^) and intima-to-media ratios were calculated as reported [21,22].

### Proliferation and apoptosis of rat carotid arteries treated with PD168077 after balloon injury

Cell proliferation and apoptosis are important contributors to neointimal formation after balloon injury. We performed experiments investigating the role of PD168077 in reducing the proliferation and sustaining the apoptosis of rat carotid artery at day14 after balloon injury. PCNA (1:500) and cleaved caspase 3 (1:800) expression were evaluated the proliferation or apoptosis respectively by western-blot as previously.

### Statistical analysis

The data are expressed as mean ± SEM. Comparison within groups was made by repeated measures ANOVA (or paired *t*^-^test when only 2 groups were compared), and comparison among groups (or *t*-test when only 2 groups were compared) was made by factorial ANOVA with Holm-Sidak test. A value of *P* < 0.05 was considered significant.

## Results

### D_4_ receptor in artery

**Figure 1:**
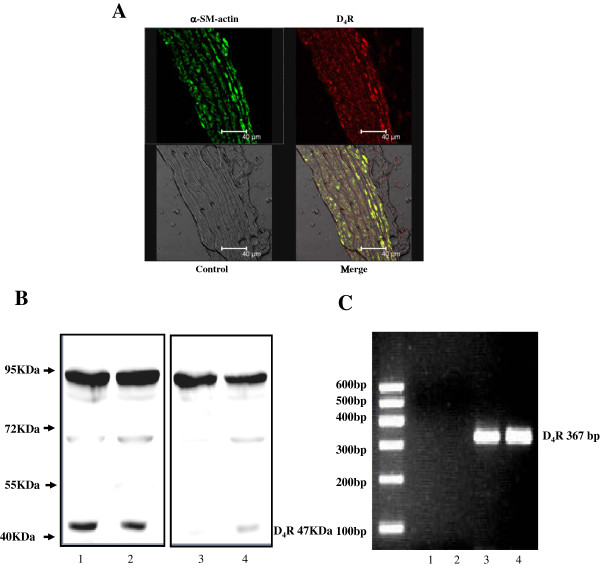
**Expression of D**_**4 **_**receptors in rat thoracic aorta and A10 cells. A**: D_4_ receptor protein expression was determined by confocal microscopy in thoracic aorta from SD rats. The arteries were washed, fixed, and immunostained for D_4_ receptor and α-smooth muscle (SM)-actin as described in the Methods. Colocalization appears as yellow after merging the images of fluorescein isothiocyanate-tagged D_4_ receptor (red) and Alexa 568-tagged α-SM-actin (green). **B**: D_4_ receptor protein expression was determined in artery by immunoblotting. The 47 kDa band indicated the expression of D_4_ receptor protein (lane1 and lane2) in A10 cells and rat thoracic aorta because it was no longer visible when the antibodies were pre-adsorbed by the immunizing peptide (1/10 w/w, lane3 and lane4). **C**. D_4_ receptor mRNA expression was determined by RT-PCR. The 367 bp band indicated the expression of D_4_ receptor mRNA in A10 cells and in rat thoracic aorta (lane3 and lane4) because the band was not observed in the absence of template (lane1) or primers (lane2).

D_4_ receptor expression in rat thoracic aorta was determined by double-immunostaining, immunoblotting, and RT-PCR analysis. The D_4_ receptor is expressed in the vascular smooth muscle, proved by its colocalization with α-SM-actin (Figure 1A). Immunoblotting revealed that a 47 kDa band, expected molecular size for the D_4_ receptor protein, disappeared when the antibody was pre-adsorbed with the corresponding immunizing peptide (1/10 w/w) (Figure 1B). RT-PCR for the D_4_ receptor in rat thoracic aorta and A10 cells showed the expected D_4_ receptor size of 367 bp (Figure 1C).

### Stimulation of the D_4_ receptor inhibits insulin-mediated A10 cell proliferation and migration

**Figure 2:**
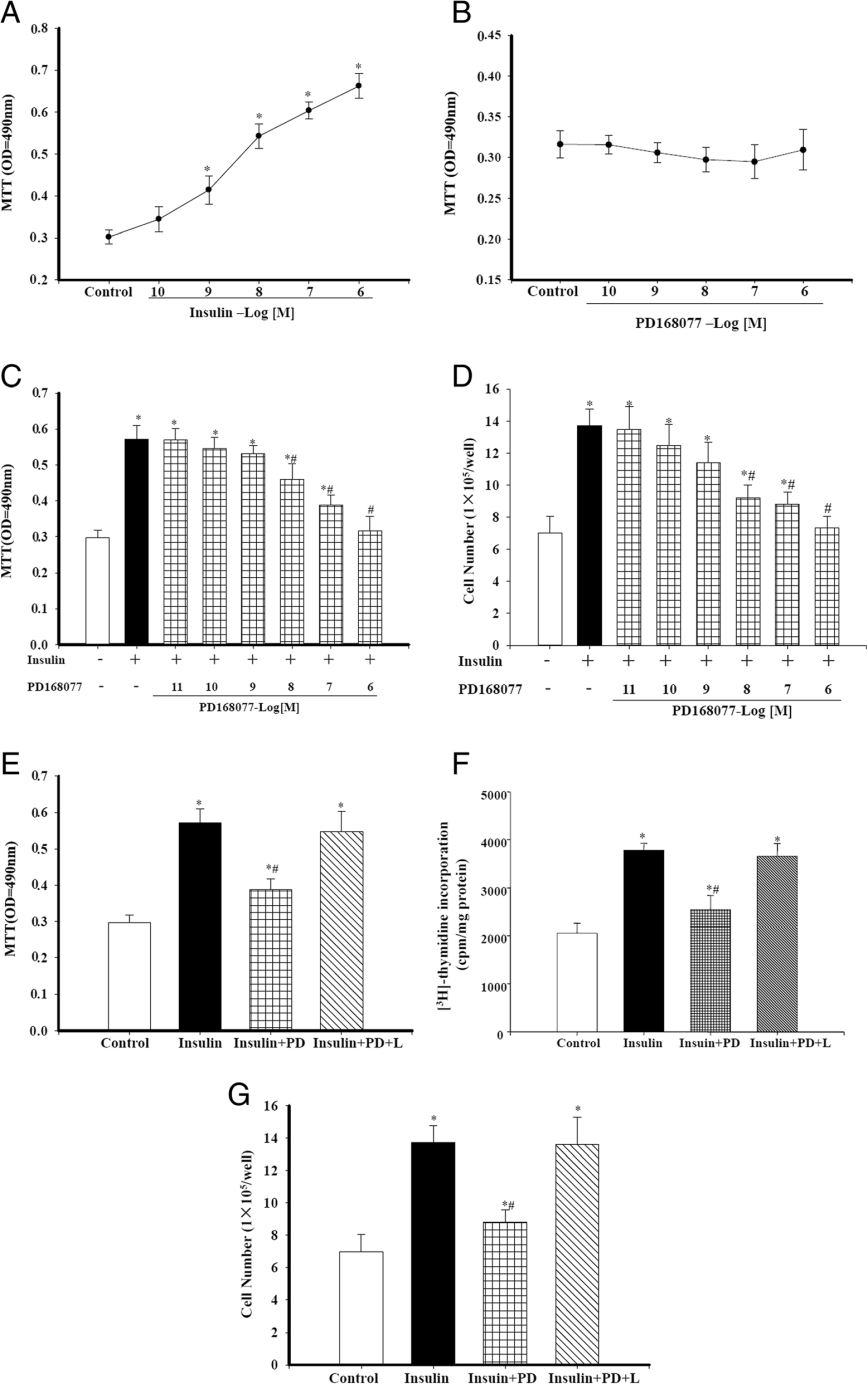
**Effect of D**_**4 **_**receptor on insulin-mediated proliferation in A10 cells. A**. Effect of insulin on the proliferation of A10 cells. A10 cell proliferation was determined by the uptake of the tetrazolium salt, 3-(4,5-dimethylthiazol-2-yl)-2,5 diphenyltetrazolium bromide (MTT) after incubation with different concentrations of insulin. Results are expressed as MTT optical density (n = 10, *P < 0.05 vs. control, one-way ANOVA, Holm-Sidak test). Control cells were treated with vehicle. **B**. Effect of D_4_ receptor agonist on the proliferation of A10 cells. A10 cell proliferation was determined by MTT after incubation with the indicated concentrations of the D_4_ receptor agonist PD168077 (10^-10^ mol/L~10^-6^ mol/L). Results are expressed as MTT optical density (n = 10, P = NS, one-way ANOVA, Holm-Sidak test). Control cells were treated with vehicle. **C** and **D**. Effect of D_4_ receptor on insulin-mediated proliferation in A10 cells. A10 cell proliferation was determined by MTT **(C)** or number of cells **(D)** after incubation with insulin (10^-7^ mol/L) in the absence or presence of varying concentrations of the D_4_ receptor agonist, PD168077 (PD, 10^-11^ mol/L - 10^-6^ mol/L). Results are expressed as MTT optical density or cell number/well (n = 8-9, *P < 0.05 vs. control; ^#^*P* < 0.05 vs. insulin alone). **E**-**G**. The specificity of D_4_ receptor action on proliferation of A10 cells. A10 cells were incubated with vehicle (control), insulin 10^-7^ mol/L alone or in the presence of the D_4_ receptor agonist, (PD, PD168077, 10^-7^ mol/L) (insulin + PD) or both D_4_ receptor agonist (PD) and D_4_ receptor antagonist (L, L745870, 10^-7^ mol/L) (insulin + PD + L) for 24 hours. A10 cell proliferation was determined by MTT **(E)** or [^3^H]-thymidine incorporation **(F)** and number of cells **(G)**. Results are expressed as MTT optical density (n = 8) or cpm/mg protein (n = 4) 0r cell number/well (n = 9; *P < 0.05 vs. control; ^#^*P* < 0.05 vs. insulin alone).

VSMCs are the main cellular components of neointimal lesions after vascular injury. Their migration and proliferation contribute to the formation of these lesions. Therefore, the effect of D_4_ receptor on A10 cell proliferation was explored by using MTT, [^3^H]-thymidine incorporation and direct cell counting. Insulin increased A10 cell proliferation in a concentration-dependent manner in A10 cells (Figure 2A). Although PD168077 by itself had no effect on cell proliferation (Figure 2B), it inhibited the insulin-mediated proliferation in A10 cells in a concentration-dependent manner (10^-8^-10^-6^ mol/L) (Figure 2C and D). The inhibitory effect of D_4_ receptor was specific because the D_4_ receptor antagonist L745870 (10^-7^ mol/L/24 hrs) blocked the cell proliferative effect of PD168077 (Figure 2E, F and G).

**Figure 3:**
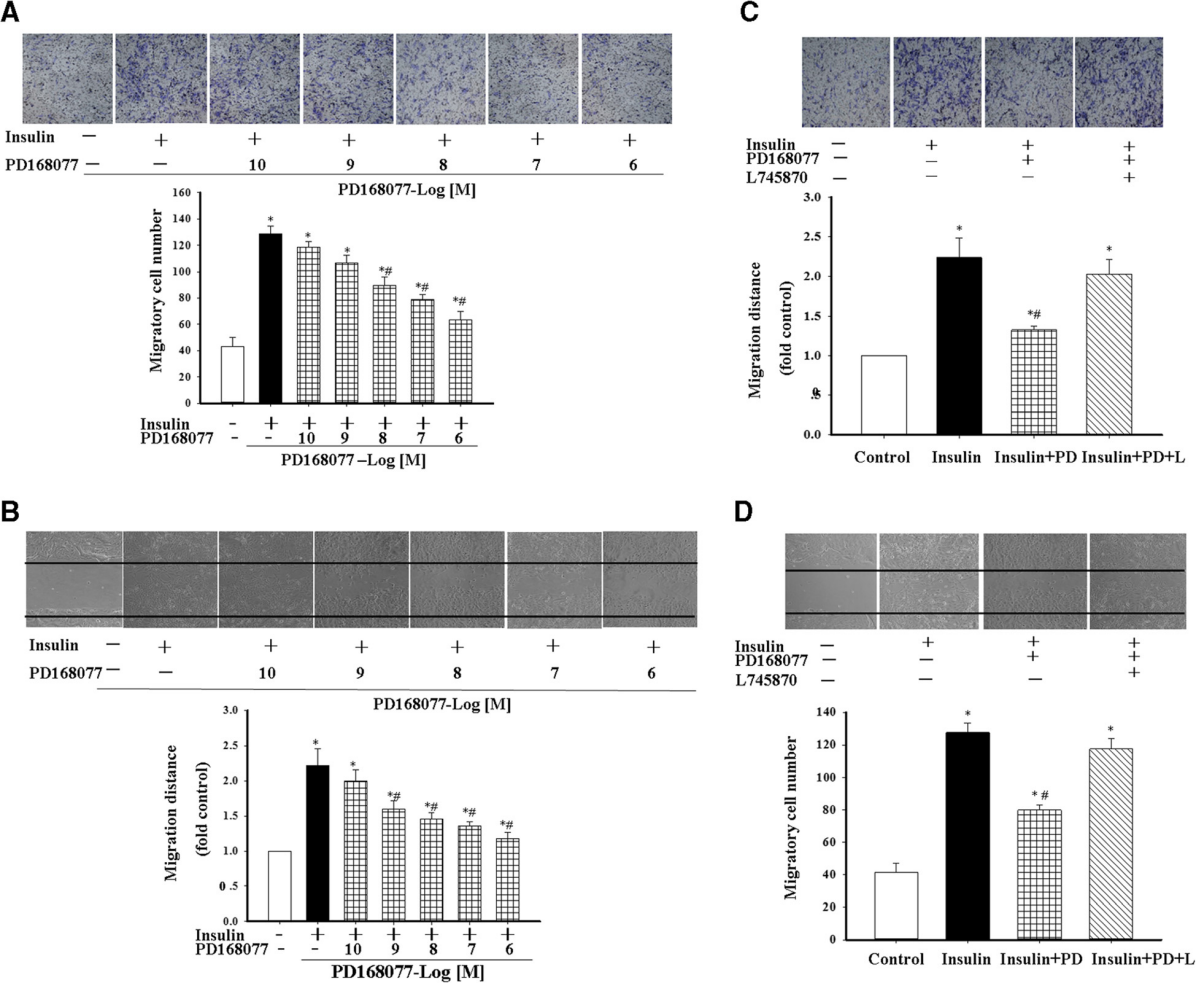
**Effect of D**_**4 **_**receptor on insulin-mediated A10 cell migration. A** and **B**. Effect of the D_4_ receptor agonist PD168077 on insulin-mediated A10 cell migration. A10 cell migration was determined by transwell **(A)** and scratch-wound migration assay **(B)** after incubation with insulin (10^-7^ mol/L), alone, or in the presence of varying concentrations of PD168077 (PD, 10^-10^ mol/ - 10^-6^ mol/L). Results are expressed as migratory cell number or migration distance (n = 9, *P < 0.05 vs. control; ^#^*P* < 0.05 vs. insulin alone). **C** and **D**. Specificity of D_4_ receptor action on insulin-mediated migration of A10 cells. A10 cells were incubated with vehicle (control), insulin 10^-7^ mol/L alone or in the presence of the D_4_ receptor agonist, (PD, PD168077, 10^-7^ mol/L) (insulin + PD) or both D_4_ receptor agonist (PD) and D_4_ receptor antagonist (L, L745870, 10^-7^ mol/L) (insulin + PD + L). A10 cell migration was determined by transwell **(C)** and scratch-wound migration assay **(D)**. Results are expressed as migratory cell number or migration distance (n = 9, *P < 0.05 vs. control; ^#^*P* < 0.05 vs. insulin alone).

We also studied the effect of insulin on cell migration by transwell and scratch-wound migration tests. Insulin increased the migration of A10 cells, an effect that was inhibited by a D_4_ receptor agonist, PD168077, in a concentration-dependent manner (Figure 3A and B). Consistent with the cell proliferation study, the inhibitory effect of PD168077 was specific to the D_4_ receptor because the D_4_ receptor antagonist L745870 (10^-7^ mol/L/24 hrs) blocked the effect of PD168077 on cell migration (Figure 3C and D).

### Activation of D_4_ receptor decreases insulin receptor expression in A10 cells

**Figure 4:**
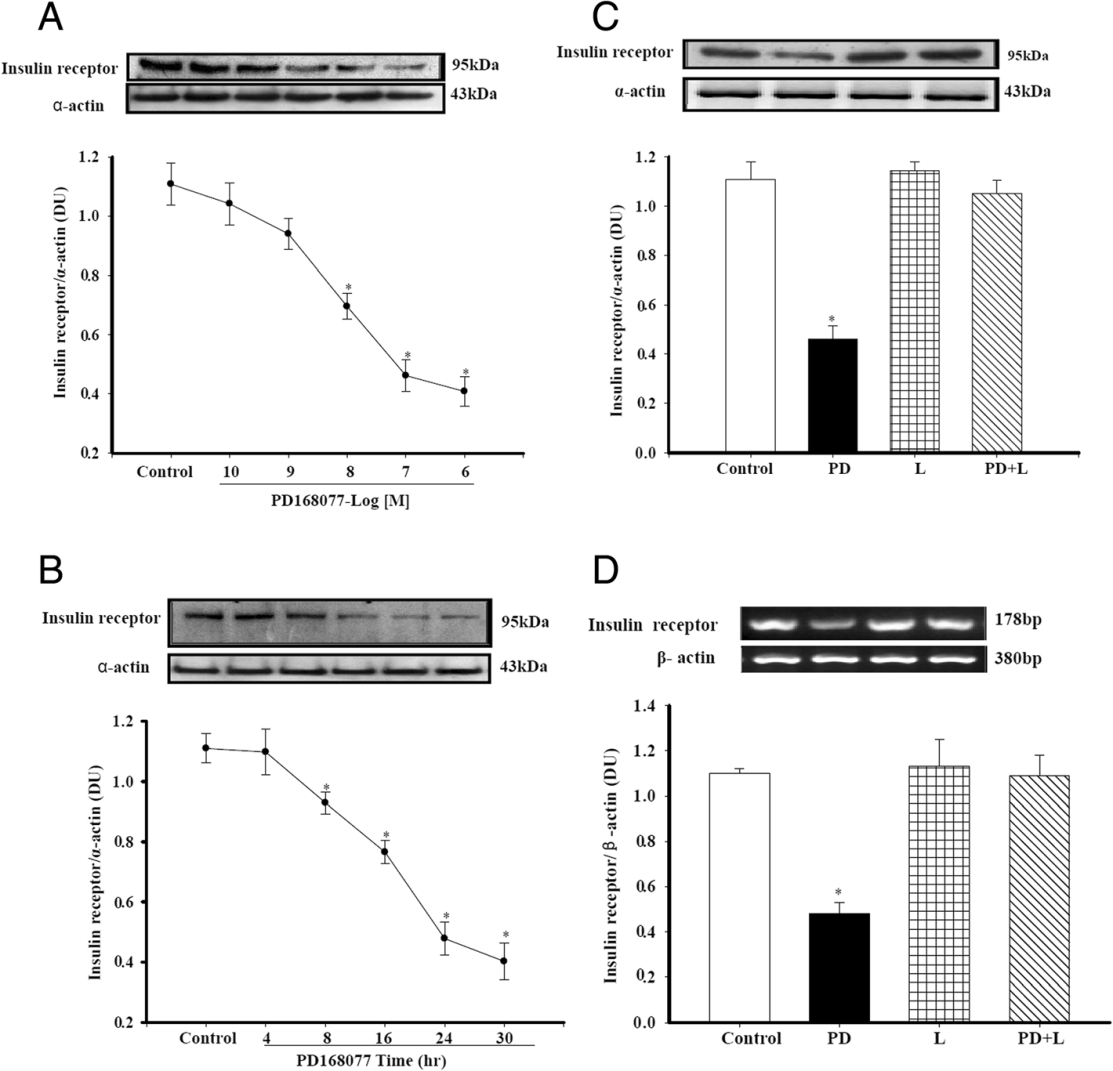
**Effect of D**_**4 **_**receptor on insulin receptor expression in A10 cells. A**. Concentration-dependent effect of the D_4_ receptor agonist PD168077 on insulin receptor protein expression in A10 cells. A10 cells were incubated with insulin in the absence or presence of varying concentrations of the D_4_ receptor agonist PD168077 (PD, 10^-10^ mol/L - 10^-6^ mol/L) for 24 hrs. Insulin receptor expression was determined by immunoblotting. Distilled water (dH_2_O), instead of insulin, was used as control. Results are expressed as the ratio of insulin receptor to α-actin densities (n = 5, *P < 0.05 vs. control). **B**. Time-course of the effect of the D_4_ receptor agonist PD168077 on insulin receptor protein expression in A10 cells. The cells were incubated at the indicated times with PD168077 (PD, 10^-7^ mol/L). dH_2_O, instead of insulin, was used as control. Results are expressed as the ratio of insulin receptor to α-actin densities (n = 5, *P < 0.05 vs. control). **C** and **D**. Specificity of D_4_ receptor effect on insulin receptor expression in A10 cells. A10 cells were incubated with the indicated reagents: (dH_2_O [Control], PD, PD168077, 10^-7^ mol/L, L,L745870, 10^-7^ mol/, or PD + L) for 24 hrs. The insulin receptor protein or mRNA expressions were determined by immunoblotting **(C)** or RT-PCR **(D)**. Results are expressed as the ratio of insulin receptor (as a fraction of 100%) to α-actin for immunoblotting or β-actin densities for RT-PCR (as a fraction of 100%) individually (n = 5, *P < 0.05 vs. others).

To uncover the mechanisms underlying the negative regulation of the D_4_ receptor of insulin-mediated proliferation and migration in A10 cells, we quantified the expression of insulin receptor. We found that stimulation of the D_4_ receptor by a D_4_ receptor agonist, PD168077, decreased insulin receptor expression in a concentration (10^-10^ -10^-6^ mol/L) and time (4-30 hrs)-dependent manner (Figure 4A and B). The effect of PD168077 on insulin receptor expression was via the D_4_ receptor, because a D_4_ receptor antagonist, L745870 (10^-7^ mol/L/24 hrs), blocked the inhibitory effect of PD168077 on insulin receptor expression in A10 cells (Figure 4C). Stimulation of the D_4_ receptor PD168077 (10^-7^ mol/L/24 hrs) also decreased insulin receptor mRNA expression, an effect that was blocked by the D_4_ receptor antagonist L745870 (Figure 4D), suggesting that the regulation of insulin receptor by the D_4_ receptor occurs at the transcriptional or post-transcriptional levels.

### Effect of D_4_ receptor on neointima formation in balloon-injured carotid arteries of SD rats

**Figure 5:**
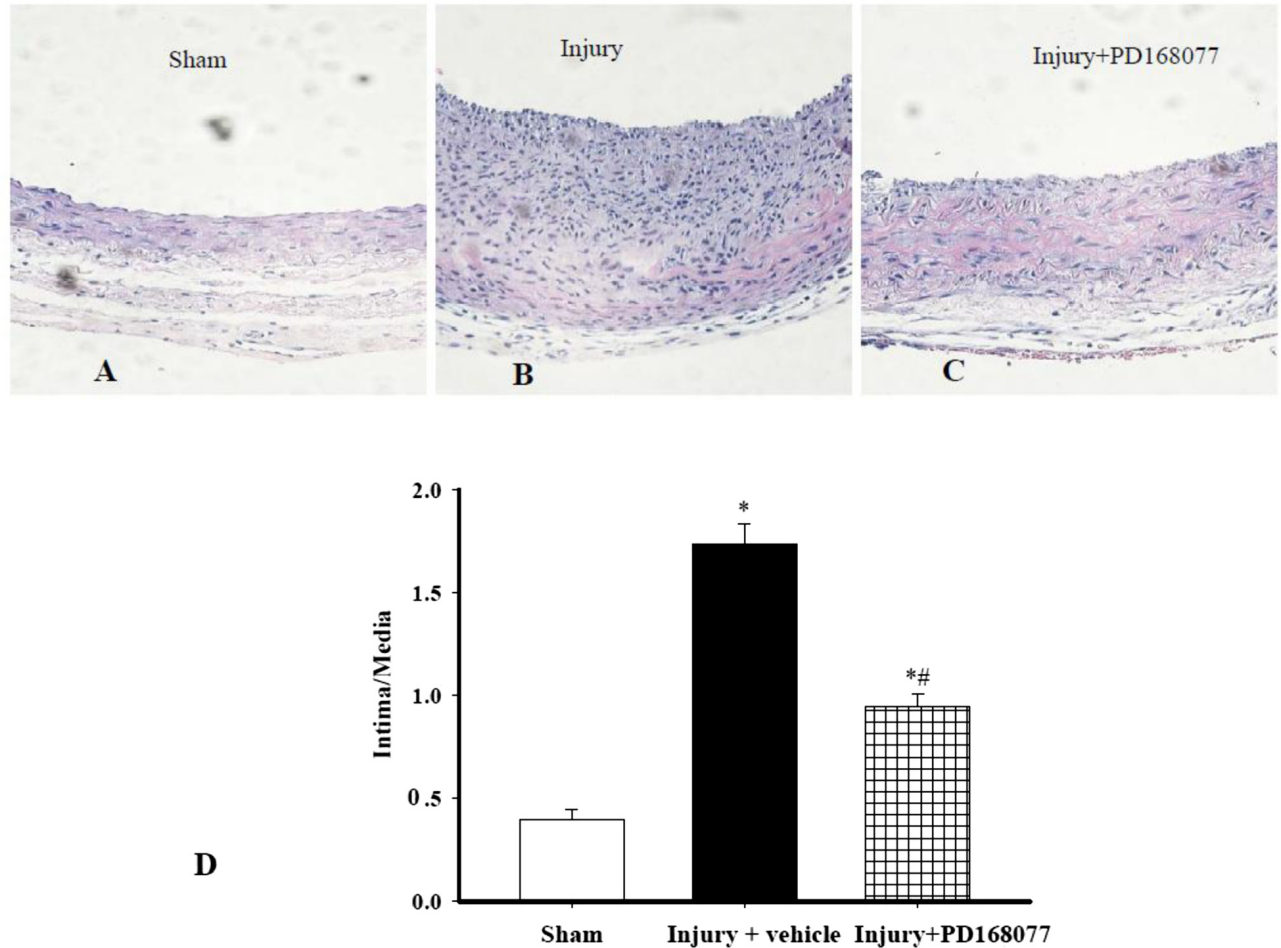
**Effect of PD168077 on neointimal formation after vascular injury in SD rats with hyperinsulinemia.** Carotid balloon-injured rats were intraperitoneally injected with vehicle or PD168077 (6 mg/kg/day) for 14 days. Representative histologies **(A-C)** were shown above the bar graphs. **A**: sham operation control. **B**: balloon-injured carotid artery and vehicle injection. **C**: balloon-injured carotid artery and PD168077 injection. The bar graphs show the area of the intima to media ratio (I/M ratio, **D**) (n = 6, *P < 0.05 vs. sham group, ^#^*P* < 0.05 vs. balloon-injured and vehicle group).

To determine whether or not the D_4_ receptor-mediated inhibition of insulin-induced proliferation and migration of A10 cells had physiological significance, we studied the effect of the D_4_ receptor agonist PD168077 in hyperinsulinemic type 2 diabetic SD rats (Table 1). A significant increase in the body weight was observed in the rats fed with HFD compared to rats fed with normal pellet diet (NPD). Before STZ injection, HFD-fed rats had similar glucose concentrations to NPD-fed rats, but significantly higher serum insulin, triglyceride, total cholesterol, and free fatty acids concentrations, Injection of STZ (25 mg kg^-1^), The level of blood glucose significantly increased compared to HFD-fed rats and NPD-fed rats, thus suggesting producing type 2 diabetes with insulin resistance. However, the blood pressure remained unaltered among STZ + HFD-treated rats, HFD-treated rats and control NPD-fed rats. We found no significant changes in each parameter between PD168077-treated and untreated STZ + HFD rats. The carotid artery-injured rats were then intraperitoneally injected with PD168077 (6 mg/kg/d) for 14 days to investigate the role of PD168077 in neointimal formation in vivo. Consistent with previous reports [21,23], a significant increase in neointimal formation was found 2 weeks after the balloon injury of the carotid artery (Figure 5A and B). Compared with balloon injury and vehicle-injected group, PD168077 significantly inhibited neointimal formation, associated with a decrease in the intima/media (I/M) ratio (Figure 5C and D).

**Table 1:** Metabolic characteristics and biochemical parameter in SD rats induced using high-fat feeding in combination with STZ and PD168077

	**NPD**	**HFD**	**HFD±STZ**	**HFD±STZ+PD168077**
Bodyweight (g)	302±7.8	344±12.8*	346±10.51*	342±10.77*
Mean blood pressure (mmHg)	60.74± 7.92	6 1.20± 8.85	64.05 ± 8.70	59.85 ±9.45
Blood glucose (mmol/L)	4.35 ±0.47	5.22 ±0.26	12.76±0.88*^#^	11.3 1±0.75*^#^
Serum insulin (pmol/L)	22.37± 7.44	68.23 ± 3.26*	77.25 ±3.87*	78.46±3.61*
Triglycerides (mmol/L)	1.33±0.39	1.98±0.11*	2.15±0.11*	2.03±0.15*
Total cholesterol (mmol/L)	2.88 ±0.47	4.55 ±0.22*	4.68±0.37*	4.69±0.35*
Free fatty acids (mmol/L)	0.26± 0.12	0.56±0.02*	0.58±0.06*	0.64±0.05*

*P < 0.05 versus NPD 7 weeks*;*^*#*^*P* < 0.05 versus HFD; NPD: nomal-pellet diet; HFD: high-fat diet; STZ: streptozotocin; PD168077: D_4_ receptor agonist; n = 6.

### Effect of D_4_ receptor on proliferation and apoptosis of in balloon-injured carotid arteries of SD rats

**Figure 6:**
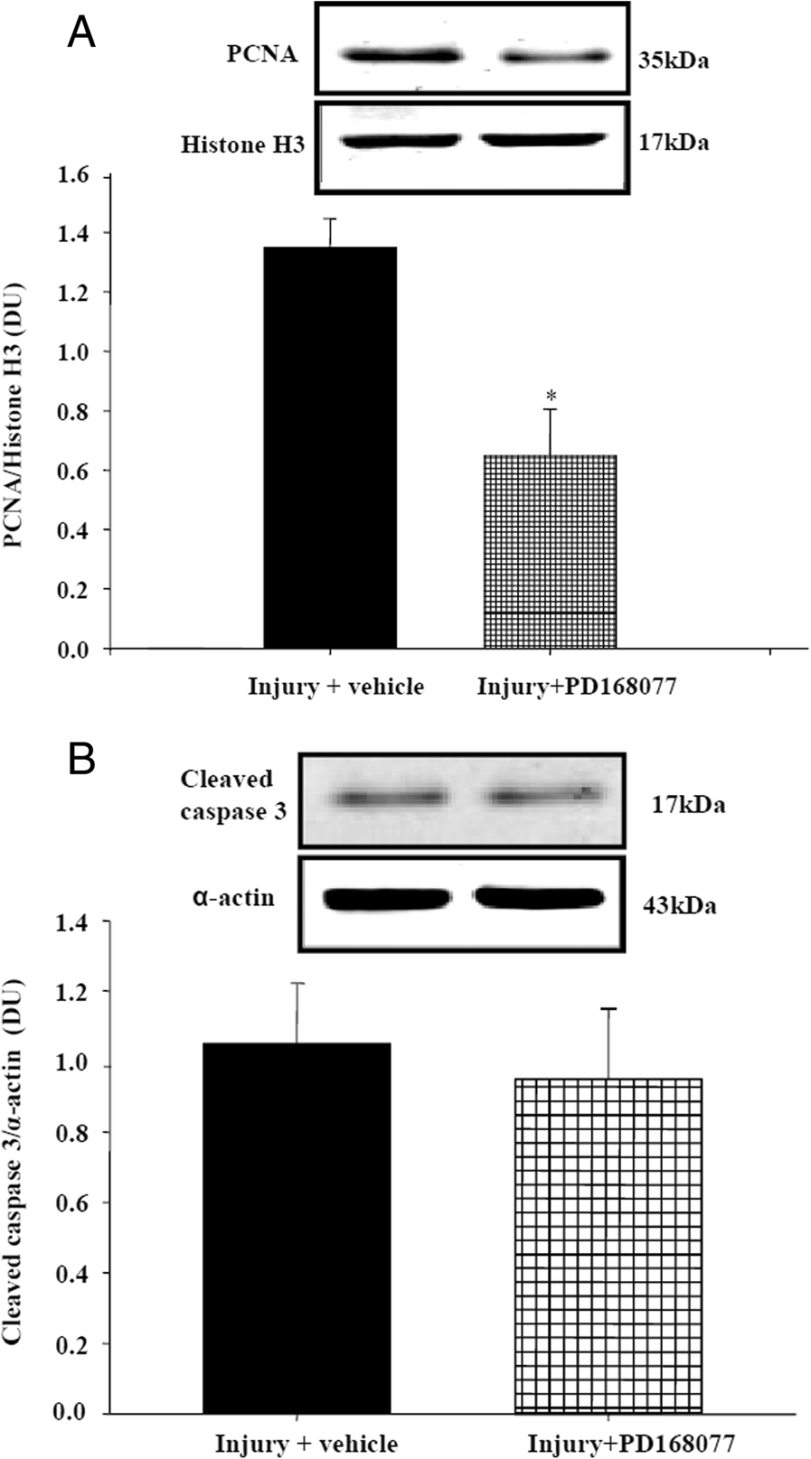
**Effects of PD168077 on the proliferation and apoptosis of balloon-injured carotid in SD rats with hyperinsulinemia.** Carotid balloon-injured rats were intraperitoneally injected with vehicle or PD168077 (6 mg/kg/day) for 14 days. Proliferation was measured by immunoblotting of proliferating cell nuclear antigen (PCNA) **(A)** and cleaved caspase 3 expression **(B)** in PD168077-treated and control rats. Results are expressed as the ratio of PCNA to Histone H3 densities or ratio of caspase-3 to α-actin densities (n = 4, *P < 0.05 vs. control).

PCNA expression revealed a significant decrease in proliferation in the PD168077-injected rats compared with vehicle-injected rats (Figure 6A). There was no difference in cleaved caspase 3 expression between two groups (Figure 6B), it seemed likely that the mechanisms of PD168077 inhibited neointimal hyperplasia were attributed to its antiproliferative and not apoptotic effects in balloon injured carotid artery of STZ + HFD rats.

## Discussion

Insulin resistance and hyperinsulinemia are important risk factors for cardiovascular disease due, in part, to the VSM proliferative and pro-atherogenic actions of insulin [3,5,24,25]. Therefore, reducing the stimulatory effect of insulin on VSMC proliferation and migration may provide new insights in the prevention of atherosclerosis.

More and more pieces of evidence indicate an interaction between insulin and dopamine receptors in various tissues and cells. The renal dopaminergic system is impaired in hyperinsulinemic rats and type 2 diabetes patients [26-28]. The dopamine D_1_-like receptor agonist, fenoldopam, has been shown to improve peripheral insulin sensitivity in STZ-induced type 2 diabetic rats [29]. In pancreatic beta cells, the D_2_-like receptor (D_2_R/D_3_R) agonist quinpirole inhibits glucose-stimulated insulin secretion [30]. Another D_2_-like receptors agonist, bromocriptine, has been reported to decrease insulin levels of obese women [31]. Our previous studies also indicate a negative interaction between D_1_-like and insulin receptors because a D_1_-like receptor agonist, fenoldopam, inhibits the proliferative effect of insulin in A10 cells [13]. Stimulation of the D_3_ receptor also inhibits insulin receptor mRNA and protein expressions and insulin-mediated VSMC proliferation [14]. However, whether or not the D_4_ receptor has inhibitory effect on insulin-mediated VSMC proliferation and migration is unknown.

Previous studies have demonstrated that D_4_ receptors are expressed in the adventitia and adventitia-media border of human pulmonary and coronary arteries [32,33]. We now show, for the first time, that D_4_ receptors are expressed in thoracic aorta of SD rats, determined RT-PCR and immunoblotting and in VSM of the aorta, by double-immunofluorescence staining. Moreover, the D_4_ receptor in VSMCs is functional. Stimulation of the D_4_ receptor reduced insulin-mediated VSMC proliferation and migration, although D_4_ receptor, by itself, had no effect.

The importance of the D_4_ receptor on VSMC remodeling during insulin resistance was investigated using a rat model of insulin resistance and hyperinsulinemia (STZ-treated rats on high fat diet). Treatment of these rats with PD168077, a D_4_ receptor agonist, for two weeks prevented neointimal growth in balloon-injured carotid artery. Neointimal formation after vessel injury is a complex process involving the proliferation and migration of VSMCs [21]. Apoptosis is also important in this process [34,35]. In this study, PD168077 significantly reduced the balloon injury caused neointimal hyperplasia in rat carotid artery. PCNA expression was also decreased in injured artery with PD168077 treatment. However, the expression of cleaved caspase 3 was not significant change in injured carotid arteries between the two groups which seems proliferation not apoptosis likely to be the major contributor for attenuation of neointimal formation after treatment with PD168077. Based on these findings and previous studies, we speculate that the D_4_ receptor may be a target to inhibit abnormal VSMC growth induced by insulin, which may lead to a novel strategy in the treatment of atherosclerosis.

In summary, we have demonstrated that activation of the D_4_ receptor inhibits insulin receptor expression and insulin-mediated proliferation and migration of A10 cells in vitro, and neointimal formation in vivo. This study implicates a critical role of the D_4_ receptor in the pathogenesis of vascular remodeling and this new insight may result in the development of a novel strategy in the treatment and prevention of vascular injury associated with insulin resistance.

## Competing interests

The authors declare that they have no competing interests.

## Authors’ contributions

CQY and ZW performed most of the experiments and analyzed data and wrote the manuscript. YH and CYC performed the animal experiments. YKL and WEW reviewed and edited the manuscript. PAJ edited the manuscript and contributed to the discussion. HYW and CYZ designed the experiments and wrote and edited the manuscript. All authors read and approved the final manuscript.
